# Kremen1 regulates mechanosensory hair cell development in the mammalian cochlea and the zebrafish lateral line

**DOI:** 10.1038/srep31668

**Published:** 2016-08-23

**Authors:** Joanna F. Mulvaney, Cathrine Thompkins, Teppei Noda, Koji Nishimura, Willy W. Sun, Shuh-Yow Lin, Allison Coffin, Alain Dabdoub

**Affiliations:** 1 Biological Sciences, Sunnybrook Research Institute, 2075 Bayview Ave, Toronto, ON, M4N 3M5, Canada; 2 College of Arts and Sciences and Department of Integrative Physiology and Neuroscience, Washington State University, Vancouver, WA, USA; 3 Department of Surgery, School of Medicine, University of California San Diego, La Jolla, CA, USA; 4 Department of Otolaryngology - Head and Neck Surgery, Department of Laboratory Medicine and Pathobiology, University of Toronto, Toronto, Canada; 5 Department of Laboratory Medicine and Pathobiology, University of Toronto, Toronto, Canada

## Abstract

Here we present spatio-temporal localization of Kremen1, a transmembrane receptor, in the mammalian cochlea, and investigate its role in the formation of sensory organs in mammal and fish model organisms. We show that Kremen1 is expressed in prosensory cells during cochlear development and in supporting cells of the adult mouse cochlea. Based on this expression pattern, we investigated whether Kremen1 functions to modulate cell fate decisions in the prosensory domain of the developing cochlea. We used gain and loss-of-function experiments to show that Kremen1 is sufficient to bias cells towards supporting cell fate, and is implicated in suppression of hair cell formation. In addition to our findings in the mouse cochlea, we examined the effects of over expression and loss of Kremen1 in the zebrafish lateral line. In agreement with our mouse data, we show that over expression of Kremen1 has a negative effect on the number of mechanosensory cells that form in the zebrafish neuromasts, and that fish lacking Kremen1 protein develop more hair cells per neuromast compared to wild type fish. Collectively, these data support an inhibitory role for Kremen1 in hair cell fate specification.

The sensory epithelium of the cochlea is a highly ordered structure comprising mechanosensory hair cells and their associated supporting cells. During development, multiple waves of signaling including Wnt, Notch, FGF, BMP and hedgehog signaling act in combination to define the prosensory region and assign cell identity^1^. In mouse, starting on embryonic day (E) 12, cells of the presumptive sensory epithelium exit mitosis progressively, from the apex of the developing cochlea towards the base ending by E13.5^2^. On E13.5 prosensory cells begin to be segregated between cells specified to become hair cells or supporting cells, and differentiate in a basal-to-apical gradient along the length of the cochlea thus forming the sensory epithelium^2^.

Disruption of canonical Wnt signaling, either through loss of β-catenin or pharmacological inhibition of Wnt signaling contemporaneous with specification of the prosensory region, resulted in failure of hair cells to differentiate^3^,^4^. When Wnt signaling was activated during formation of the sensory epithelium, the post mitotic prosensory cells re-entered mitosis and more hair cells were observed^3^,^4^. The pro-mitotic effect of β-catenin was also observed in early postnatal cochleae^5^,^6^. While this demonstrates that Wnt signaling is involved in cell fate specification and regulation of the cell cycle, these phenotypes did not pinpoint which cells were endogenously responsive to Wnt signaling; every β-catenin expressing prosensory cell was affected. In order to determine how secreted Wnts pattern the developing sensory epithelium, it is necessary to investigate location and function of Wnt receptors and their modulators.

Kremen1 is a single pass transmembrane protein that acts as a receptor to members of the dickkopf (Dkk) family of Wnt antagonists^7^. Kremen1 functions as part of a Wnt inhibitory complex that prevents Lrp5/6 mediated sequestration of Gsk3β^7^, allowing it to target cytoplasmic β-catenin for degradation. On receipt of a Dkk ligand Kremen1 associates with Lrp5/6, removing it from the cell surface via clathrin mediated endocytosis, thus attenuating Wnt signal transduction^7^,^8^,^9^.

Based on previous reports that place Kremen1 atop the Wnt cascade acting to inhibit Wnt binding to its cogent receptors, we examined the effects of manipulating receptor composition on Wnt responsive cells of the cochlea. Through gain and loss-of-function experiments, we show that Kremen1 is involved in regulation of cell fate decisions in the mammalian cochlea and the zebrafish lateral line.

## Results

### Kremen1 is expressed in the developing mouse cochlea

**Figure 1:**
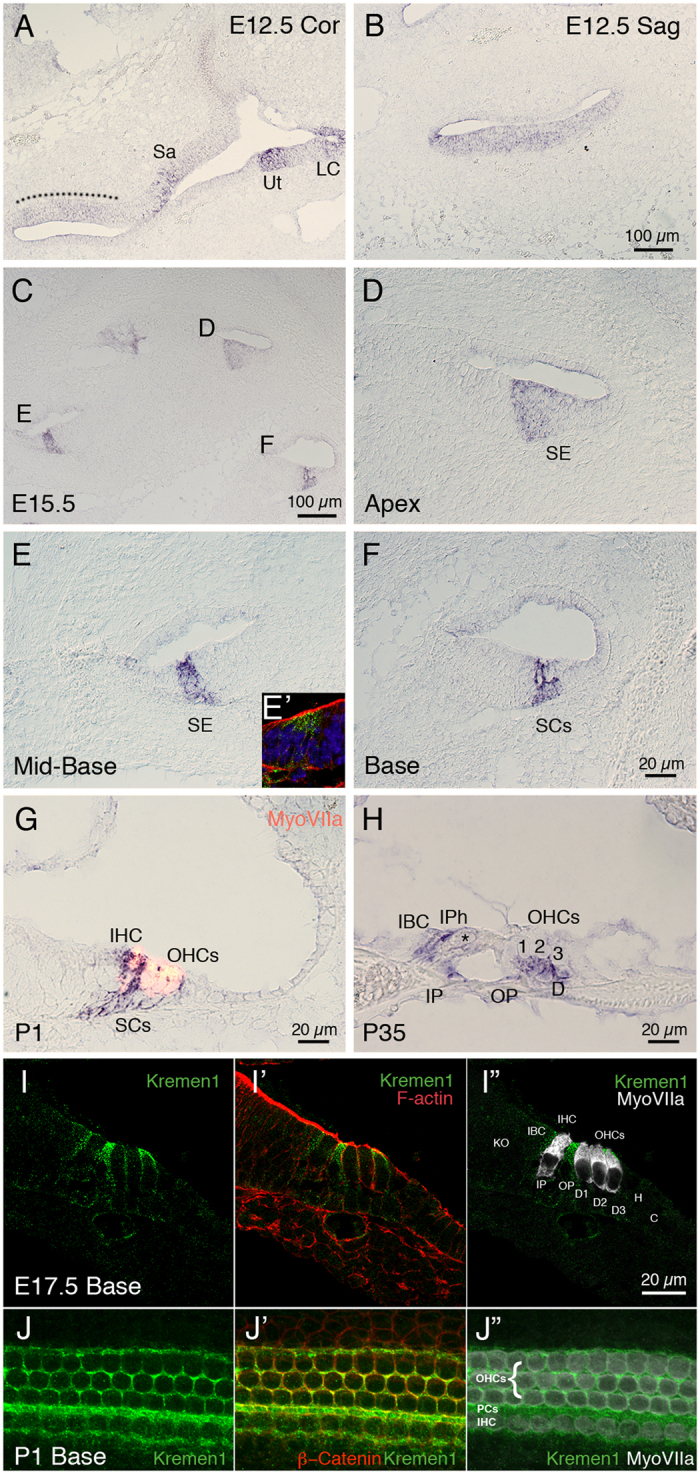
*Kremen1* is expressed in the developing and adult mammalian cochlea. (**A**) Coronal section through the head at E12.5. *Kremen1* expression is detected in the floor of the cochlear duct (dotted line) and prosensory region of the vestibular system. (**B**) A sagittal section through the head at E12.5, *Kremen1* expression is found in the floor of the cochlear duct. (**C**) A low magnification view of a transverse section through an E15.5 cochlea showing *Kremen1* expression in all turns. (**D**) *Kremen1* expression in the prosensory region of the apical turn of an E15.5 cochlea. (**E**) *Kremen1* expression in the prosensory region of the midbasal turn of an E15.5 cochlea. (**E’**) Immunohistochemistry for Kremen1 (green), cell membrane (actin, red) and cell nuclei (Dapi, blue) in the midbasal turn on E15.5. Kremen1 protein localized to the prosensory region. (**F**) *Kremen1* expression in the supporting cell region of the base of an E15.5 cochlea (D, E, scale as in panel F). (**G**) High magnification view of transverse sections through P1 cochlea showing *Kremen1* expression in supporting cells. Hair cells are counterstained for MyoVIIa, in red. (**H**) High magnification view of transverse section through P35 cochlea. (**I,I’,I”**) High magnification view of transverse section through E17.5 cochlea showing immunohistochemistry for Kremen1 (green), cell membrane (actin, red and hair cells (MyoVIIa, white). (**J,J’,J”**) High magnification view of the luminal surface of the basal region of a P1 cochlea showing immunohistochemistry for Kremen1 (green), cell membrane (β-Catenin, red) and hair cells (MyoVIIa, white). SE; sensory epithelium, Sa; saccule, Ut; utricle, LC; lateral canal, IHC; inner hair cell, OHC; outer hair cell, IBC; inner border cell, IP; inner pillar cell, IPh; inner phalangeal cell, OP; outer pillar cell, PCs; Pillar cells, D; Deiters’ cells, SC; supporting cell.

Using reverse transcriptase PCR, we determined that *Kremen1* and Dkk family members *Dkk2, Dkk3* and *Dkkl1* were expressed in the cochlear duct on embryonic (E) day 12.5 and E15.5, coincident with formation of the sensory epithelium (Table 1). We have previously reported that Dkk3 is expressed in the greater epithelial ridge throughout cochlear development and maturation^10^. We observed that Dkk1 was expressed at low levels on E15.5, but was not detected on E12.5 (consistent with previous data we reported in Geng *et al*.^10^), and that Dkk4 and Kremen2 were not detected in the developing cochlea on E12.5 or E15.5 (Table 1). We then localized *Kremen1* expression using *in situ* hybridization and immunohistochemistry. On E12.5, when the prosensory region is in the early stages of specification, *Kremen1* was expressed in the floor of the duct (Fig. 1A,B). The sensory region of the mammalian cochlea has a developmental gradient such that the developing base of the cochlea is more mature than the developing apex. Sagittal sectioning through the cochlea on E15.5 allowed visualization of the developing sensory epithelium at different developmental stages (Fig. 1C). By E15.5, in the least developmentally advanced region, the apex (Fig. 1C,D), and in the midbase (Fig. 1C,E), expression was restricted to the prosensory region. In the most developmentally advanced region of the cochlea, the base, *Kremen1* expression was only localized to the supporting cell region (Fig. 1C,F). On postnatal day 1 (P1), when the developmental process is almost complete, we observed *Kremen1* expression only in inner border cells, inner phalangeal cells, pillar cells, and Deiters’ cells (Fig. 1G). We did not detect expression in hair cells. This restricted expression pattern was also observed in adult mice (Fig. 1H). Kremen1 protein was localized to the apical region of supporting cell plasma membrane (Fig. 1I,I’). These results suggest that *Kremen1* is continuously expressed in the supporting cells and is implicated in patterning and maintenance of the sensory epithelium.

### Over expression of Kremen1 in the mouse sensory epithelium has an inhibitory effect on hair cell formation

**Figure 2:**
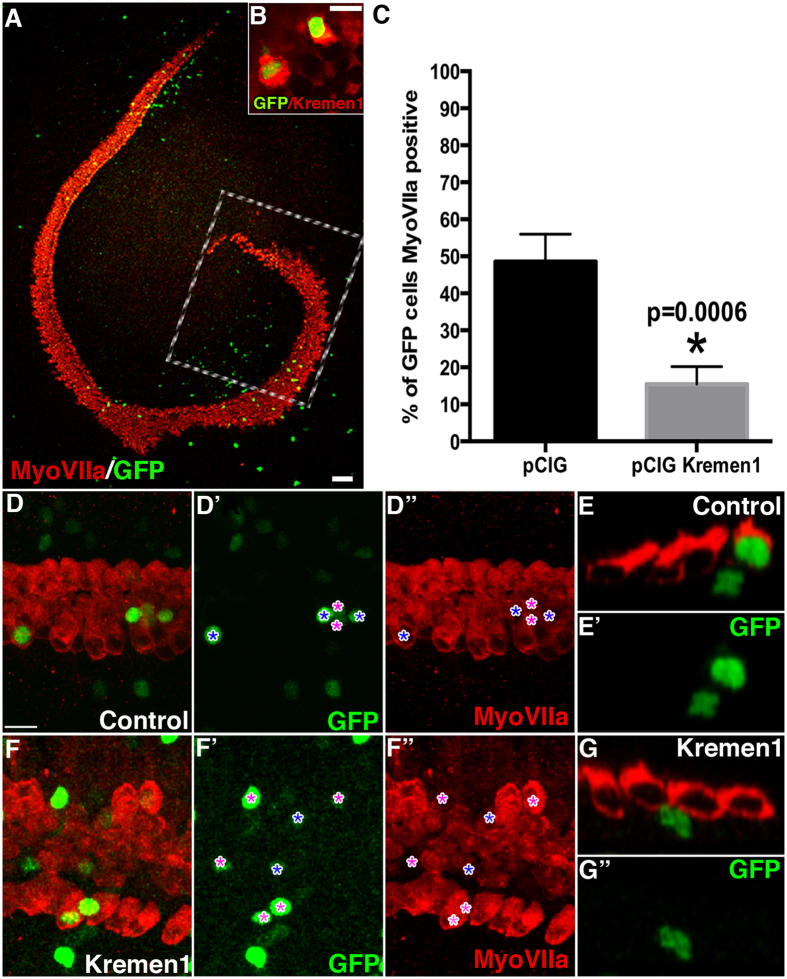
Kremen1 over expression had an antagonistic effect on hair cell fate. (**A**) A low magnification view of a typical cochlear explant electroporated on E13.5 with a GFP transfection reporter (GFP). Explant cultures were established and electroporated on E13.5 and cultured *in vitro* for six days. Hair cells are labeled with antiMy oVIIa (red). Boxed region represents the apical portion of the duct where cells were quantified. Scale indicates 100 μm. (**B**) High magnification view of two cochlear cells transfected with *pCIG.Kremen1.IRES.nucGFP* (green) in the Kremen1 expressing region and stained by polyclonal anti Kremen1 (red), indicating that our expression construct generated robust levels of *Kremen1*. Scale indicates 10 μm. (**C**) Mean percentages of transfected cells that expressed MyoVIIa. Asterisk indicates p < 0.05. (**D,D’,D”**) High magnification surface view of apical region of a cochlear explant electroporated with empty vector. GFP positive cells are marked by asterisks (*) in blue if the GFP positive cell was colabeled by antiMyoVIIa, or purple if not. Scale indicates 20 μm. (**E,E’**) High magnification orthogonal view of a cochlear explant electroporated with empty vector. Two GFP positive cells are shown in close proximity within the sensory epithelium. One cell sits bellow the hair cell layer (in the supporting cell level) and one cell is colabelled with MyoVIIa. (**F,F’,F”**) High magnification surface view of apical region of a cochlear explant electroporated with *pCIG.Kremen1.IRES.nucGFP*. GFP positive cells are marked by asterisks (*) as in (**D**). (**G**) High magnification orthogonal view of a cochlear explant electroporated with *pCIG.Kremen1.IRES.nucGFP*.

Should Kremen1 inhibit Wnt signaling in the cochlea, we hypothesized that introduction of excess Kremen1 would prevent hair cell differentiation. We tested this hypothesis by expressing ectopic Kremen1 in the prosensory epithelium. Plasmid containing the Kremen1 coding sequence (*pCIG.Kremen1.IRES.nucGFP*) or empty plasmid (*pCIG.nucGFP*) was electroporated into E13.5 cochlear explants and cultured *in vitro* for 6 days. Explant cultures were fixed and labeled for the hair cell marker MyoVIIa. Analysis was restricted to the most apical 25% portion of the cochlear duct (Fig. 2A), targeting prosensory cells prior to hair cell and supporting cell differentiation. GFP positive cells were counted within the sensory epithelium and assigned as hair cells based on expression of MyoVIIa (Fig. 2C–G). Of control plasmid electroporated cells, in total 49% differentiated as hair cells (Fig. 2C–E) while significantly fewer cells (15%) transfected with Kremen1 plasmid developed as hair cells (Fig. 2C,F,G, Table 2, p = 0.0006).

We also examined the influence of *Kremen1* over expression on proliferation. Cochlear explants were harvested and electroporated with Kremen1 plasmid on E13.5 and cultured *in vitro* for 48 hours before addition of BrdU conditioned media. Explants were maintained for a further 72 hours and then fixed for analysis. GFP positive non-sensory cells of the lesser epithelial ridge within 200 μm of the sensory epithelium were counted and assigned as having undergone cell division if they contained nuclear BrdU. In the non-sensory region, there was no significant difference in the number of double labeled BrdU and GFP positive cells in the control or Kremen1 expressing cells (Table 3). Kremen1 over expression also did not activate proliferation in the sensory region. We observed no BrdU positive GFP positive cells in the sensory epithelium in either control transfected or *pCIG.Kremen1.IRES.nucGFP* transfected explants.

### Knock down of Kremen1 biases prosensory cells towards hair cell fate

**Figure 3:**
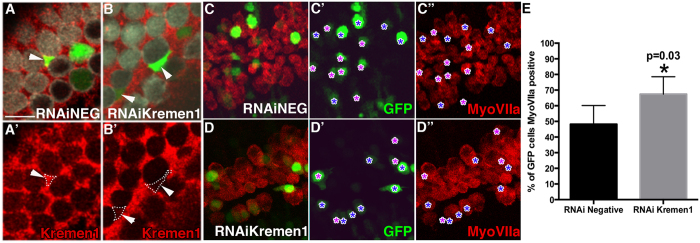
Loss of Kremen1 made a prosensory cell more likely to develop as a hair cell. (**A,B**) High magnification surface view of negative control RNAi construct (**A**) or *Kremen1RNAi* (**B**) transfected cochlear explant, hair cells are labeled by antiMyoVIIa (white), supporting cells are labeled by antiKremen1 (red), transfected cells express GFP (green). (**A**) Arrowhead indicates a supporting cell transfected with *RNAiNeg* construct that expresses Kremen1 on its surface at similar levels to non transfected neighboring cells. (**A’**) Single channel view of (**A**). (**B**) Arrowheads indicate supporting cells transfected with K*remen1RNAi* that display very little Kremen1 protein staining relative to their non-transfected neighbors. (**B’**) Single channel view of (**B**). Scale bar indicates 10 μm. (**C,D**) Surface view of apical region of a cochlear explant electroporated on E12.5 and cultured *in vitro* for six days, with either negative control RNAi (**C,C’,C”**) or *Kremen1RNAi* (**D,D’,D”**). Hair cells are labeled by antiMyoVIIa (red), transfected cells express GFP. Purple asterisks (*) show GFP cells that do not express MyoVIIa, blue asterisks mark GFP positive cells that express MyoVIIa. (**E**) Quantification of transfected cells expressing MyoVIIa. Asterisk denotes p < 0.05. All error bars represent standard deviation.

Having established that Kremen1 has an inhibitory effect on hair cell development, we tested whether loss of Kremen1 would promote hair cell fate. An RNAi construct targeted against *Kremen1* was generated and tested for efficacy by electroporating cochlear explants on E15.5. Supporting cells transfected with RNAi negative control construct strongly expressed Kremen1 protein (Fig. 3A) while supporting cells expressing *Kremen1RNAi* had lower levels of Kremen1 compared to surrounding non-transfected supporting cells (Fig. 2B) indicating that Kremen1 protein expression was down regulated in transfected cells.

We targeted the apical prosensory region of the cochlea on E12.5 to determine whether cells expressing *Kremen1RNAi* were more likely to assume a hair cell fate. We selected E12.5 in order to allow down regulation of Kremen1 prior to commitment of sensory cell fate. Cochleae were dissected and electroporated either with *Kremen1RNAi* construct or with negative control RNAi construct. Hair cells were labeled with antiMyoVIIa. GFP positive (RNAi expressing) cells were quantified in the apical quarter of the sensory epithelium and assigned as hair cells based on colabelling with antiMyoVIIa. In cultures electroporated with the negative control RNAi construct, 48% of GFP positive cells differentiated into hair cells, while 67% of cells expressing *Kremen1RNAi* differentiated into hair cells (Fig. 2C–E, Table 2, p = 0.03). As development progressed, loss of Kremen1 had less influence on cell fate. Targeting prosensory cells on E13.5 resulted in 40% of RNAi negative control transfected cells taking a hair cell fate, while 60% of *Kremen1RNAi* transfected cells became hair cells (Table 2, p = 0.01). RNAi transfection on E15.5, showed a further reduction in cell fate determination, with 24% of RNAi negative control transfected cells becoming hair cells, versus 36% of *Kremen1RNAi* transfected cells, unlike at E12.5 and E13.5, this difference was not statistically significant (Table 2, p = 0.25).

We also tested whether loss of Kremen1 protein would allow proliferation in cells of the postmitotic prosensory region. Cochlear explants were harvested and electroporated with negative control or *Kremen1RNAi* on E12.5 and maintained in BrdU conditioned media for six days. *Kremen1RNAi* transfected cells were no more likely to exhibit BrdU incorporation than cells expressing the negative control (Table 3, p = 0.36).

### Kremen1 is involved in determining the number of hair cells contained within a zebrafish neuromast

**Figure 4:**
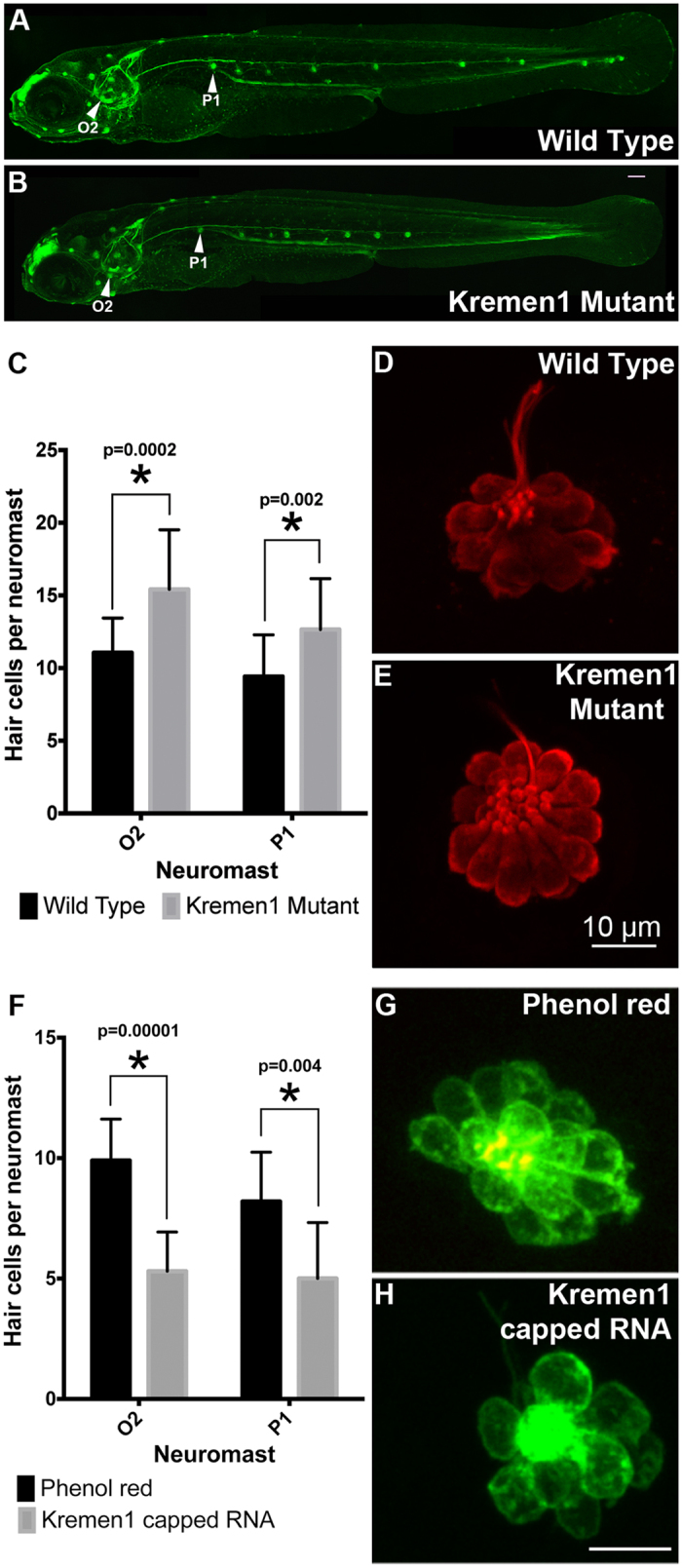
Kremen1 modulates hair cell number in zebrafish neuromasts. (**A**) A lateral view of a whole mount fish expressing *Tg(cldnB:GFP)*. This fish has wild type distribution of neuromasts. Neuromasts O2 and P1 are labelled. (**B**) A lateral view of a whole mount fish mutant for *Kremen1* and expressing *Tg(cldnB:GFP)*. This fish has fewer neuromasts in the posterior tail region. Neuromasts O2 and P1 are labelled. Images provided by Drs H. McGraw and A. Nechiporuk. (**C–E**) Kremen1 mutants have excess hair cells per neuromast. (**C**) Mutants have a significant increase in hair cells in neuromasts O2 and P1. Asterisk (*) indicates p < 0.005. Hair cells (Parvalbumin, red) in neuromast O2 of a wild type fish (**D**) or a *Kremen1* mutant fish (**E**). (**F–H**) Kremen1 over expression reduces hair cell numbers. (**F**) Fish injected with Kremen1 capped RNA have fewer hair cells per neuromast than control (phenol red) injected fish, asterisk (*) denotes p < 0.005. Hair cells (GFP) in neuromast P1, of a control injected (**G**) or Kremen1 capped RNA injected (**H**) fish. Scale bar indicates 10 μm. All error bars represent standard deviation.

To examine the influence of Kremen1 on hair cell development in neuromasts of the lateral line in zebrafish, we analyzed a transgenic Kremen1 mutant line (Krm1^nl10^ allele) generated on a Tg(cldn:GFP) background^11^. In agreement with previous reports^11^, the mutant fish had fewer neuromasts (Fig. 1A,B). Consistent with our previous work on activation of Wnt signaling in the lateral line^12^, we focused our analysis on neuromasts O2 and P1 (location indicated in Fig. 1A,B). Hair cells (defined by expression of the hair cell marker parvalbumin) within each neuromast were quantified (Fig. 4C–E). We observed significantly more hair cells in the O2 and P1 neuromasts in 5 day post fertilization (dpf) Kremen1 mutants than in age-matched wildtype animals (Kremen1 mutant: O2, 15.42 ± 4.10; P1, 12.66 ± 3.50. Wildtype: O2, 11.10 ± 2.38; P1, 9.41 ± 2.87), (O2, p = 0.0002; P1, p = 0.002, two tailed t-test). These data concur with previously observed reports of Kremen1 inhibition at position O2^13^.

We then examined whether over expression of Kremen1 inhibited hair cell formation. Brn3c:mGFP transgenic fish, which express membrane-bound GFP in all hair cells, were injected with phenol red (vehicle control) or 350 ng *Kremen1* mRNA. We observed that there was a statistically significant reduction in the number of hair cells in fish injected with Kremen1 mRNA, compared with age-matched controls (Kremen1 injected O2, 5.3 ± 1.63; P1, 5.0 ± 2.32; wildtype, O2, 9.9 ± 1.73; P1, 8.2 ± 2.04) (O2, p = 0.0001; P1, p = 0.04, Fig. 4F–H).

## Discussion

We report that in the embryonic mouse cochlea, Kremen1 was expressed first in the prosensory domain and that *Kremen1* expression was restricted to supporting cells as development proceeded. Over expression of Kremen1 had a significant inhibitory effect on the differentiation of hair cells, while reduction in the levels of Kremen1 made a prosensory cell more likely to take a hair cell fate. These data are consistent with an antagonistic effect on canonical Wnt signaling, as Wnt signaling is required for hair cell formation^3^,^4^.

Previous studies have revealed that β-catenin is necessary for hair cell differentiation and that stabilization of β-catenin in all prosensory cells leads to supernumerary hair cells and prosensory cell proliferation^3^,^4^. Given that current literature reports that Kremen1 is an inhibitory component of the Wnt signaling network, our results suggest that canonical Wnt signaling takes place in cells fated to become hair cells, and that Kremen1 prevents receipt of this signal in cells fated to become supporting cells. This effect was limited to cells that had not yet been committed to a hair cell or supporting cell fate; as the cochlea matured the ability of Kremen1 knock down to induce hair cell fate was reduced. We speculate that on E15.5, the hair cells and supporting cells had been specified, and knock down of Kremen1 had no significant effect.

Furthermore, by reducing expression of Kremen1 on E12 prior to the onset of hair cell differentiation, we found that while transfected cells were more likely to differentiate as hair cells, they did not undergo cell division. The lack of a proliferative response when Kremen1 was downregulated indicated that should Kremen1 normally inhibit canonical Wnt signaling in the ear, endogenous levels of Wnt signaling were not sufficient to return post mitotic cells to a proliferative state in the absence of Kremen1. It is more likely that if endogenous Wnt signaling in the sensory region of the cochlea is modulated by Kremen1, it would control cell fate decisions, rather than cell cycle check points in the prosensory cells E13.5 onwards. This does not preclude a role for canonical Wnt signaling induced proliferation prior to E12.5 or in the periotic mesenchyme.

Kremen1 reportedly acts by disrupting the composition of Wnt receptors and co-receptors on the cell membrane^7^. Supporting cells maintain expression of members of the Wnt receptor complex throughout development, but do not proliferate or change fate unless exogenous factors are applied or β-catenin signaling is activated intracellularly^3^,^4^. Wnt signal transduction is repressed, likely either by the presence of secreted Wnt antagonists, or the absence of key members of the Wnt receptor complex. We demonstrated that Dkk1, Dkk2, Dkk3 and Dkkl1 are expressed in the cochlear duct during development of the cochlea (Table 1), suggesting that Dkk family of secreted Wnt antagonists are putative ligands for Kremen1. The presence of three out of five members on E12.5 and four out of five on E15.5 indicates that there is likely functional redundancy between these ligands. Membrane bound components of Wnt signaling, including Frizzled receptors, Lrp co-receptors and Wnt potentiating Lgr receptors, are expressed in supporting cells during development and at neonatal stages^5^. Frizzleds 1, 2 and 4 are expressed in both hair cells and supporting cells^5^; expression of Frizzled receptors is necessary in hair cells after differentiation in order to transduce non-canonical Wnt signaling to orientate stereociliary bundles^14^. Lgr receptors function to allow secreted Rspondin molecules to potentiate Wnt signaling by retaining members of the Frizzled/Lrp5/6 receptor complex at the cell surface^15^. While Lgr receptors are expressed in supporting cells, supporting cells respond modestly to stimulation with Rspondin1^16^; this could be because Kremen1 blocks Rspondin function by removing Lrp5/6. The ability of a given cell to respond to Wnt signaling is dependent on the combination of receptors that it expresses. We place Kremen1 on the cell surface of prosensory cells and then supporting cells, and we show that Kremen1 expression biases cells away from a hair cell fate; together this indicates that Kremen1 acts to disrupt the transduction of hair cell inducing signaling events.

In zebrafish, Kremen1 over expression also had a negative effect on hair cell formation. Over expression of Kremen1 led to a reduction in the number of hair cells per neuromast, while loss of Kremen1 protein resulted in an increase in the number of hair cells per neuromast, but a decrease in the number of neuromasts at the posterior end of the trunk lateral line. It has previously been proposed that Kremen1 inhibits proliferation of prosensory cells located in deposited neuromasts since morpholino-mediated knock down of Kremen1 resulted in an increase in neuromasts size, consistent with our results in the krm1^nl10^ mutant^13^. However, loss of Kremen1 inhibited proliferation of neuromast precursor cells in the migrating lateral line primordium by increasing levels of free Dkk1b and Dkk2^11^, resulting in the formation of fewer neuromasts^11^. It is therefore possible that in the krm1^nl10^ fish, the anterior neuromasts contain additional hair cells due to increased deposition of precursors in these neuromasts, rather than to increased proliferation post-deposition. Over expression of Kremen1 limited the number of hair cells that form in the O2 and P1 neuromasts but did not increase the number of neuromasts that were deposited. This raises the question of whether hair cell precursors are segregated between hair cell and supporting cell fate by Wnt signaling during development, whether they require Wnt signaling in order to proliferate after being deposited, or whether the distribution of hair cells between neuromasts is controlled by Wnt signaling.

Kremen1 signaling likely acts at multiple points within lateral line development, with lateral line patterning in the krm1^nl10^ fish resembling Wnt loss-of-function, rather than Wnt gain-of-function^11^. It is unlikely that the function of Kremen1 in sensory cell development recapitulates its role in migration of the posterior lateral line primordium. If Kremen1-mediated capture of Dkk ligands allowed Wnt signaling to upregulate proliferation within the deposited neuromasts, we would expect to see fewer hair cells and supporting cells per sensory organ in fish lacking Kremen1. Our experiments, and those by Wada *et al*.^13^, demonstrate that the opposite is true. Loss of Kremen1 results in an increase in the number of hair cells per neuromast, while over expression of Kremen1 resulted in fewer hair cells per neuromast. Since neuromasts are deposited and mature progressively, it is possible that Kremen1 has two distinct roles. Dkks are secreted ligands that can act at a distance from their source to inhibit Wnt signaling independently of Kremen1. Dkk expression is expanded in the migrating primordium of krm1^nl10^ fish, but expression in deposited neuromasts is unknown, nor is it known whether Dkk and Kremen1 act cooperatively in the later stages of neuromast development^11^. The role of endogenous Wnt signaling in neuromast hair cell formation is still unclear as manipulation of Dkk2^13^ and Kremen1 appear to affect hair cell number, whereas manipulation of downstream Wnt components such as Lef1 have no effect^17^,^18^. Given that Kremen1 appears necessary for both propagation and inhibition of proliferation depending on the stage of development, further study is required to clarify temporal functions of Wnt signaling during development of hair cell epithelia.

## Methods and Materials

### Animals

Mouse tissue was harvested from CD-1 mice of either sex (Charles River) maintained and euthanized in accordance with Sunnybrook Research Institute and Canadian Council on Animal Care guidelines for the care and use of laboratory animals. All mouse work was approved by Sunnybrook Research Institute Animal Care and Use Committee.

Zebrafish were maintained and euthanized in agreement with animal welfare regulations set out by the NIH. All zebrafish work was approved by the Washington State University Institutional Animal Care and Use Committee.

### Plasmids

Riboprobe template was pYXAsc-Kremen1 (Open Biosystems) cut with SalI, transcribed by T3 polymerase. Kremen1 open reading frame was cloned from pYXAsc-Kremen1 into *pCIG.IRES.nucGFP*^19^. Zebrafish Kremen1 open reading frame was cloned from pME18S-FL3-Kremen1 (NCBI Accession number: BC158176) (Open biosystems) into pCS2+. RNAi constructs were generated by cloning the following Kremen1 specific RNAi seed sequences contained in the 3′ UTR of EmGFP into *pCIG.IRES.nucGFP*.

Kremen1-TGCTGCACAGAAGCAGGCATAGCCTGGTTTTGGCCACTGACTGACCAGGCTATCTGCTTCTGTG,

Negative-GAAATGTACTGCGCGTGGAGACGTTTTGGCCACTGACTGACGTCTCCACGCAGTACATTT.

### PCR

Positive control tissue (Forelimb, E13.5; skin, E15.5; eye, E13.5) was collected and 10 cochlear ducts each (E12.5 and E15.5) were dissected out of the temporal bone and RNA harvested using Qiagen RNeasy purification kit. RNA was harvested for each tissue type on three independent occasions, and each RT PCR experiment was carried out three independent times. Reverse transcription was performed using Accupower RT pre-mix (Bioneer). PCR was performed for 35 cycles. Primers were: Kremen1 F-GTGCTTCACAGCCAACGG R-ACGTAGCACCAAGGGCTC; Kremen2 F-ACGACTAGGCATCTATGAAG, R-AAGGCACGGAGTAGGTTC; Dkk1 F-TCTGCTAGGAGCCAGTGCC, R-GATGGTGATCTTTCTGTATCC^20^; Dkk2 F- TGCCACAGTCCCCACCAAGGATC, R-CCTGATGGAGCACTGGTTTGCAG^20^; Dkk4 F-GCCCTGGTTCTGGACTTTAACAAC, R-CTGACACCTCCTGCGAACTCTAC^21^; Dkkl1 F-ACTGAGGGTCTTGCTGCTGCT, R-GGAAGTTCCTAGGAAGGTCTC^22^.

### *In Situ* hybridization

*In situ* hybridization was performed as previously described^10^.

### Immunofluorescence

Immunofluorescence was performed as previously described^23^,^24^. Phalloidin (1:1000) (Life Technologies). Antibodies: Kremen1 (1:1000; R&D Systems), MyosinVI and MyosinVIIa (1:1000, Proteus Biosciences), Sox2 (1:250, R&D Systems), BrdU with 30 minutes antigen retrieval in 1N HCl (1:250, R&D Systems), GFP (1:500, Life Technologies), anti-parvalbumin3 (1:500 EMD Millipore).

### Kremen1 gain and loss-of-function

Explant cultures and electroporations were performed as previously described^3^,^25^; BrdU was used as previously described^3^. Explant cultures were maintained *in vitro* for six days before fixation and analysis.

### Zebrafish over expression and loss-of function

Kremen1 capped RNA was generated using the mMessage Machine SP6 kit (Ambion) and purified with Ambion’s MegaClear kit. mRNA was microinjected into Brn3c:mGFP zebrafish embryos at the 1–2 cell stage. Controls were injected with 1–2% phenol red (also included in mRNA to visualize the injection site). *Kremen1* mutant zebrafish were obtained from the Nechiporuk lab at Oregon Health & Science University^11^.

### Image analysis and statistics

Bright field and epifluorescence images were captured using an Axioscope microscope fitted with an Axiocam (Zeiss). Fluorescence images were captured using a Leica SP5 or SP8 confocal microscope (Leica). Each experiment was carried out on a minimum of three independent occasions. Within each replicate, control and experimental cochlear explant electroporations were performed on litter-mate cochleae with developmental stage determined using the Theiler staging criteria^26^. All cell counts were performed using image J and statistics were performed using Graph Pad Prism statistical software (Graph Pad). In order to quantify effects on cell fate in the cochlea, we counted GFP positive cells throughout the apical quarter of the explant in the region marked by MyoVIIa (Fig. 2A). We then used orthoganol views, and scanned down through each Z-plane to ascertain whether the GFP signal was localized to the nucleus of MyoVIIa positive cells, since MyoVIIa does not stain hair cell nuclei and the GFP was bright in the nucleus. Student’s T-tests were performed on mouse and zebrafish gain and loss-of-function experiments.

## Additional Information

**How to cite this article**: Mulvaney, J. F. *et al*. Kremen1 regulates mechanosensory hair cell development in the mammalian cochlea and the zebrafish lateral line. *Sci. Rep*. **6**, 31668; doi: 10.1038/srep31668 (2016).

## Figures and Tables

**Table 1:** Tabulated results of reverse transcriptase PCR screen for Kremen1, Kremen2 and Dickkopf family members.

	Positive Control	E12.5 Cochlea	E15.5 Cochlea
Kremen1	Forelimb E13.5	+++++	+++++
Kremen2	Forelimb E13.5	−	−
Dkk1	Forelimb E13.5	−	+
Dkk2	Forelimb E13.5	+++	++++
Dkk3	Forelimb E13.5	+++++	+++++
Dkk4	Skin E15.5	−	−
DkkL-1	Eye E13.5	+++	+++

Positive control tissue was selected according to previous reports of expression^27^,^28^,^29^,^30^. Relative levels of expression are denoted by+, no expression detected is denoted by −.

**Table 2:** Tabulated results of cochlear explant experiments focusing on cell fate.

Construct	Developmental stage	Number of experiments, number of cochleae, number of GFP + ve cells	% GFP cells expressing MyoVIIa ± standard deviation	P value derived from student’s T test
pCIG.nucGFP	E13.5	4, 11,507	49 ± 7.4	P = 0.0006
pCIG.Kremen1.IRES.nucGFP		4, 9, 305	15 ± 4.7	
RNAi Negative control	E12.5	5, 18, 342	48 ± 11.95	P = 0.03
	Kremen1 RNAi	5, 15, 410	67 ± 11.15	
RNAi Negative control	E13.5	4, 15, 270	40 ± 6.3	P = 0.01
	Kremen1 RNAi	4, 12, 260	60 ± 8.6	
RNAi Negative control	E15.5	3, 10, 154	24 ± 3.67	P = 0.25
	Kremen1 RNAi	3, 11, 186	36 ± 13.04	

**Table 3:** Tabulated results of cochlear explant experiments focusing on proliferation.

Construct	Developmental Stage	Number of experiments, number of cochleae, number of GFP + ve cells	Location of cells quantified	Mean% GFP + ve cells expressing BrDU ± standard deviation	P value derived from student’s T test
pCIG.nucGFP	E13.5	3, 7, 289	Lesser Epithelial Ridge	57 ± 18.2	P = 0.67
pCIG. Kremen1.IRES.nucGFP		3, 6, 386		50 ± 20.4	
RNAi Negative control	E12.5	3, 11,451	Entire organ of Corti	3.7 ± 3.6	P = 0.36
Kremen1 RNAi		3, 7, 366		1.3 ± 1.9	
